# Prevalence and Socioeconomic Disparities of Cigar Use in China: Findings from the China Health Literacy Survey with a Focus on the ‘Knowledgeable but Economically Marginalized’ (KEM) Population

**DOI:** 10.3390/healthcare13060583

**Published:** 2025-03-07

**Authors:** Yi Liu, Yinghua Li, Xin Xia, Zhao Liu, Zheng Su, Rui Qin, Ying Xie, Zhenxiao Huang, Anqi Cheng, Xinmei Zhou, Jinxuan Li, Xiaowen Wei, Qingqing Song, Liang Zhao, Dan Xiao, Chen Wang

**Affiliations:** 1 Department of Tobacco Control and Prevention of Respiratory Disease, Center of Respiratory Medicine, China-Japan Friendship Hospital, Beijing 100029, China; ly_2022@126.com (Y.L.); xiaxiaoxin123@163.com (X.X.); lzlzq2004@126.com (Z.L.); suzheng@zryhyy.com.cn (Z.S.); qinrui1594809@126.com (R.Q.); xieying1110@zju.edu.cn (Y.X.); zhenxiaoh1994@163.com (Z.H.); chenganqi531@126.com (A.C.); zhouxinmei_zr@163.com (X.Z.); lijinxuan1993@163.com (J.L.); weixiaowen20@163.com (X.W.); lareina_sq@163.com (Q.S.); zhaoliang0817@126.com (L.Z.); wangchen@pumc.edu.cn (C.W.); 2 School of Population Medicine and Public Health, Peking Union Medical College, Chinese Academy of Medical Sciences, Beijing 100005, China; 3 National Center for Respiratory Medicine, State Key Laboratory of Respiratory Health and Multimorbidity, National Clinical Research Center for Respiratory Diseases, Institute of Respiratory Medicine of Chinese Academy of Medical Sciences, Beijing 100029, China; 4 WHO Collaborating Center for Tobacco Cessation and Respiratory Diseases Prevention, Beijing 100029, China; 5 China Health Education Center, Beijing 100011, China; liyinghua729@sina.com; 6 School of Health Policy and Management, Peking Union Medical College, Chinese Academy of Medical Sciences, Beijing 100730, China; 7 China-Japan Friendship School of Clinical Medicine, Capital Medical University, Beijing 100029, China

**Keywords:** cigar smoking, prevalence, socioeconomic factors, China, tobacco control

## Abstract

**Background**: Cigar smoking poses significant public health challenges due to its rising prevalence and associated health risks. However, research on cigar use in China remains limited. This study investigates the prevalence, demographic characteristics, and key factors associated with cigar use among Chinese adults. **Methods**: We analyzed data from the 2018–2019 China Health Literacy Survey, including 86,701 participants aged 20–69 years. Multistage stratified sampling was employed, and logistic regression was used to estimate odds ratios (ORs) and 95% confidence intervals (CIs) for factors associated with cigar use. Weighted data were applied to ensure national representation. **Results**: Of the 86,701 respondents, 1025 participants reported having used cigars, including 248 exclusive cigar users and 777 dual users of cigars and other tobacco products. Cigar use was significantly higher among men (1.93%) than women (0.05%). Most users were aged 50–59, with a mean age of 49.3 years. Factors associated with cigar use among men included higher education (for college and higher, OR: 1.81; 95% CI: 1.42–2.30), lower household income (for income < 20,000, OR: 1.02; 95% CI: 1.08–1.53), poor self-reported health (OR: 1.45; 95% CI: 1.15–1.83), and severe nicotine dependence (FTND ≥ 7 points, OR: 2.09, 95% CI: 1.67–2.61). Cigar use prevalence showed notable regional variation, with the highest rates observed in northern and eastern provinces. **Interpretation**: The estimated number of cigar users in China is approximately 10.46 million. Male cigar users often represent a unique demographic: “knowledgeable but economically marginalized” individuals, characterized by higher education but lower economic status. Tailored tobacco control measures addressing regional disparities, socioeconomic factors, and marketing-driven misconceptions about cigars are essential to reduce public health impacts.

## 1. Introduction

Cigars are composed of chopped tobacco wrapped in tobacco leaves or a similar substance containing tobacco [1]. They come in various forms, including cigarillos and small cigars, which are increasingly marketed in ways resembling traditional cigarettes. Modern packaging and advertising strategies have made cigars more accessible, with smaller formats like cigarillos being presented in flexible packaging similar to cigarette packs [2].

A tobacco use survey covering 16 countries and approximately 3 billion people found that among the respondents, about 852 million people use tobacco, of which 572 million are cigarette users [3]. Despite global tobacco control efforts leading to a downward trend in cigarette smoking rates [4], the use of other tobacco products—such as cigars, e-cigarettes, smokeless tobacco, and waterpipe tobacco—is on the rise in certain regions. Notably, the increase in cigar sales has been particularly pronounced [5,6]. For instance, in the United States, cigar sales grew by 8.5% between 2002 and 2012, with continued growth observed in subsequent years [7]. This trend highlights the shifting landscape of tobacco consumption, where cigars have become an increasingly popular choice. The health risks associated with cigar use are well-documented, including increased mortality from cancer, cardiovascular diseases, and other conditions [8,9,10,11,12,13]. Additionally, cigar use contributes to significant economic burdens through healthcare costs and productivity losses [14].

However, studies on cigars have largely focused on Western populations, with limited research available on their prevalence and associated factors in China. A gap remains in understanding how cigars fit into the broader landscape of tobacco use in Chinese society. It has been observed that a significant proportion of individuals who utilize traditional cigarettes belong to demographics characterized by lower levels of education and economic status [15]. Conversely, a notable segment of electronic cigarette users tends to hail from populations that exhibit higher educational attainment and income brackets [16]. While cigar users in China may not conform to conventional profiles of tobacco users [17], instead, they often represent a unique demographic—individuals with higher education levels but lower economic status, a group we define as “knowledgeable but economically marginalized”.

This study aims to fill the research gap by presenting findings from the 2018–2019 China Health Literacy Survey, a nationally representative dataset. The objectives are to (1) estimate the prevalence of and total number of cigar smokers in China, (2) identify demographic and socioeconomic factors associated with cigar use, and (3) explore the unique characteristics of Chinese cigar users, particularly the interplay between education and economic status.

## 2. Method

### 2.1. Study Design and Participants

The China Health Literacy Survey (CHLS) is a nationally representative household survey of non-institutional men and women aged 15–69. Six rounds had been conducted since 2012. In the 2018 CHLS, questionnaires on smoking behavior and tobacco dependence were included for the first time, providing a unique opportunity to estimate the prevalence and associated factors of cigar smoking in China. Details of the CHLS had been reported separately [15]. The smoking questionnaire used the Global Adult Tobacco Survey (GATS) [18] and the China Adult Tobacco Survey [19].

**Figure A1:**
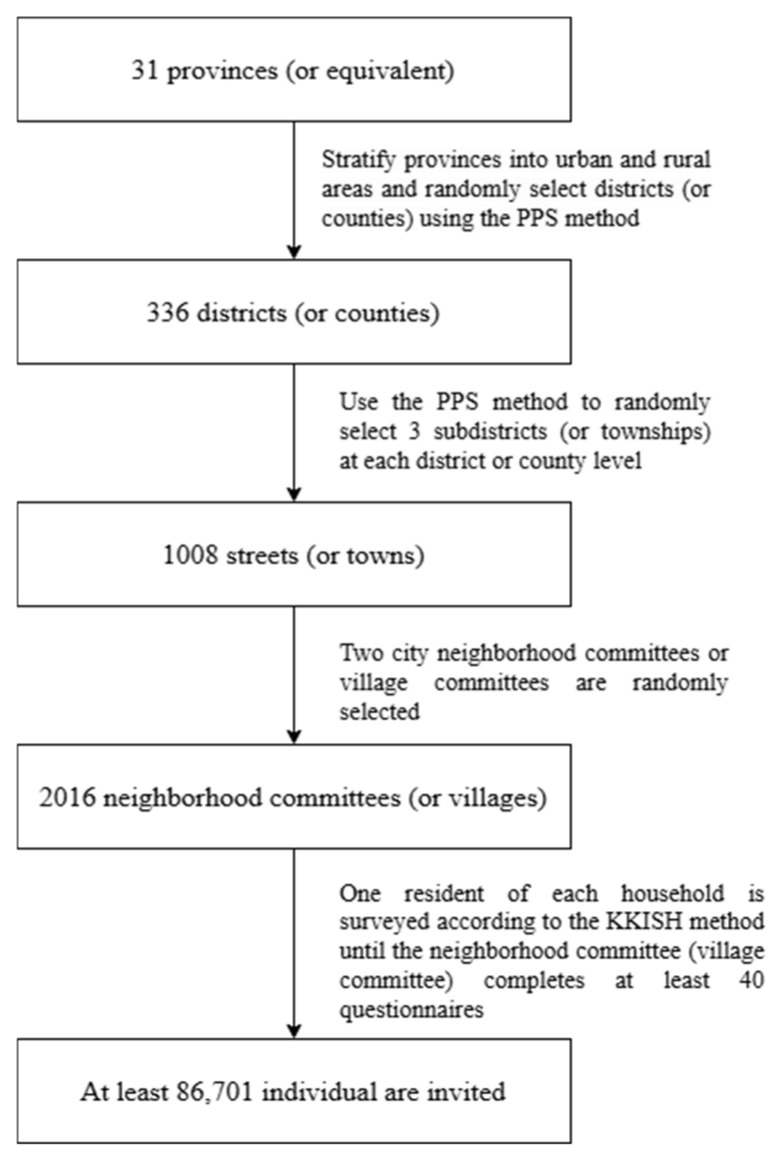
Flow diagram of study sampling procedure.

The study is based on data from the 2018–2019 China Health Literacy Survey, which employed a multistage stratified sampling approach to ensure national representation. The selection of urban and rural populations followed a probability-proportional-to-size (PPS) method based on the 2010 Chinese National Census data. We have elaborated on the post-stratification weighting procedure, which adjusts for age, gender, and urban–rural distribution to align with national demographic characteristics. The sampling procedure for the 2018 Health Literacy Survey consisted of four stages. The first stage involved the PPS method to randomly select 1008 streets or townships from 336 urban or rural areas across the 31 provinces in China. The selection of these areas was based on the population size of each district or county, which was determined using data from the Sixth National Population Census of China [20]. Then, 2016 neighborhood committees or village committees were selected from the selected streets or townships by random sampling, and 55 households were selected from each neighborhood committee or village committee selected for investigation. In short, a total of 87,708 people were invited to participate in the research survey (Appendix A Figure A1).

**Figure A2:**
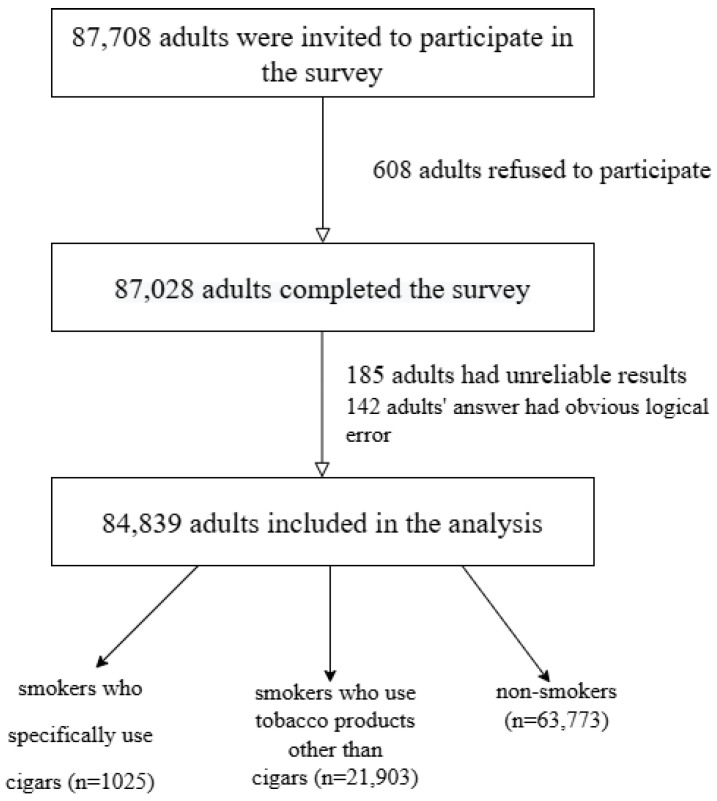
Flow diagram of participants through the study.

A total of 1007 individuals were excluded from the analysis due to providing unreliable results. The final study sample consisted of 86,701 participants, including 41,374 men and 45,327 women. Participants were categorized into three groups: non-smokers, smokers who use tobacco products other than cigars, and smokers who specifically use cigars (Appendix A Figure A2).

The study was approved by the Ethics Review Committee of the China Health Education Center (Beijing, China). All study participants provided written informed consent. The ethical approval was waivered according to Article 32 of the Measures for Ethical Review of Life Sciences and Medical Research Involving Humans in China, issued by the State Council of China in February 2023. See details in: https://www.gov.cn/zhengce/zhengceku/2023-02/28/content_5743658.htm (accessed on 20 October 2024).

### 2.2. Data Collection

For this study, cigar users were defined as people who use cigars, cigarillos, and little cigars; use of cigars was defined as having ever used any cigar product; ever used tobacco products other than cigars was defined as smokers who never use cigars.

The questionnaire collected demographic information, including age, gender, education level, and annual income. Referring to our previous study and other international studies [15], we divided the ages into five groups. Smokers were asked what type of tobacco products they used; the age at which they started smoking; the duration of smoking and the number of cigarettes smoked per day. The severity of tobacco dependence was assessed by the Fagerström Test for Tobacco dependence (FTND), which is a six-item scale and smoking addiction is divided into three levels, the total score of 0–3 is mild, 4–6 is moderate, and 7–10 is severe tobacco dependence [21].

### 2.3. Statistical Analysis

Continuous variables are presented as Mean ± SD (standard deviation), and categorical variables are presented as frequency and percentage. We analyzed the demographic characteristics of the participants and compared the demographic significant differences between the cigar-use population and those that have never used any tobacco product. ANOVA or Student’s *t*-test were employed for continuous variables, while the rank-sum test was used for continuous variables that did not meet the assumptions of parametric tests. The Chi-square test was utilized for categorical variables to assess their statistical significance.

Logistic regression analysis using different adjustment levels estimated the odds ratio (OR) and 95% confidence interval (CI) for cigar selection in the population. The independent variable included gender, age, urbanization, ethnicity, marriage status, education level, annual family income, self-reported health status and FTND score. Additionally, whether participants were cigar users or not was used as the dependent variable in the study. The model was adjusted for sex, age, urbanization, ethnicity, marriage status, education level, annual family income, self-reported health status, and FTND score.

Furthermore, we used weighted data to assess the characteristics of Chinese groups that used cigars in 2018 (the weight is calculated based on 2016 population data [20]) and estimated the proportion and amount of cigar use in different demographics, as well as their 95% CI. We also analyzed the distribution of cigar users in different provinces in China. The sample weights are calculated by using the software SUDAAN 11.0. We firstly calculated the design weight, then age and gender were included to calculate the non-response weight, and finally the sample weights were post-stratified to match the 2010 Chinese population census.

All statistical analyses were performed using SPSS 26. All reported *p*-values were two-sided, and the significance was set at *p* < 0.05.

### 2.4. Role of the Funding Source

The funders of the study had no role in study design, data collection, data analysis, data interpretation, or writing of the report. DX had full access to all the data and responsibility for the decision to submit for publication.

## 3. Result

Table 1 lists the baseline characteristics of study participants. Of the 86,701 participants included, 1025 participants reported having used cigars, with 248 using only cigars and 777 using both cigars and other tobacco products. The vast majority (95.9%) were male, with a mean age of 49.3 years (SD 12.5). Most participants were of Han ethnicity (90.3%) and nearly half (47.2%) lived in urban areas. The married population accounted for 83.7% of cigar users, and the majority (82.6%) had completed middle or high school education. Income levels were evenly distributed across the sample.

**Figure 1:**
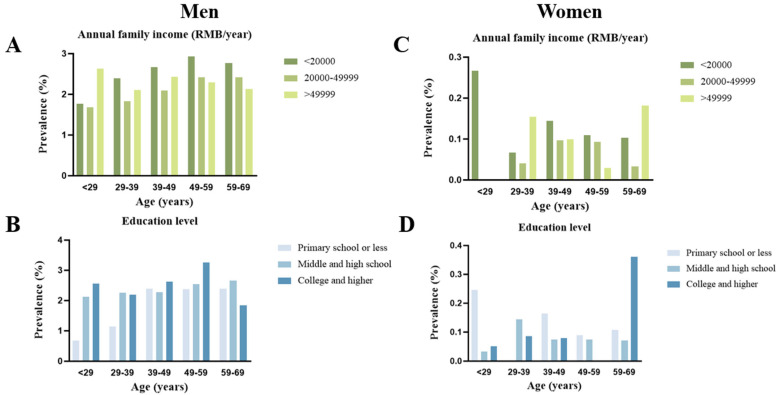
Prevalence of cigar use in Chinese adults. Note: (**A**,**B**) the prevalence of cigar users by annual family income and education level based on men; (**C**,**D**) the prevalence of cigar users by annual family income and education level based on women.

Figure 1 presents the prevalence of cigar usage among the study population, analyzed by annual household income, education level, age groups, and gender. For men, cigar usage was most prevalent (2.68%) among those with annual household incomes below 20,000 yuan, a rate significantly higher than for those earning between 20,000 and 49,999 yuan (2.19%) and those earning above 49,999 yuan (2.30%) (*p* = 0.03). This trend persisted across all age groups, with the prevalence particularly pronounced in the 39–49 and 49–59 age brackets for the lowest income category. Regarding education, the highest prevalence of cigar usage among men (2.49%) was observed in individuals with college or higher degrees, followed by those with primary school or less (2.41%) and middle or high school graduates (2.25%) (*p* < 0.0001). Notably, cigar usage peaked in the 49–59 age group among those with higher educational attainment, suggesting a potential link between educational exposure and smoking behaviors. Among women, cigar usage prevalence was considerably lower across all income categories, remaining below 0.3%. The highest rates were seen in the lowest income group (<20,000 yuan) in the younger (<29 years) and older (59–69 years) age brackets. However, the limited variability by income highlights the relative rarity of cigar use among women. In terms of education, cigar usage was similarly low. The highest prevalence was found in women aged 59–69 years with primary school education or less, while minimal cigar use was reported among women with middle or high school and higher educational levels (The detailed data supporting the key results shown in Figure A1 can be found in Appendix A).

Table 2 lists the subject-adjusted ORs for cigar use associated with baseline information among cigar users and users of tobacco product other than cigars among the study population. Multivariable-adjusted analyses showed that among men, cigar use was significantly associated with higher education (for college and higher, OR: 1.81, 95% CI: 1.42–2.30), lower annual family income (for income < 20,000 yuan, OR: 1.02, 95% CI: 1.08–1.53), poor self-reported health (OR: 1.45, 95% CI: 1.15–1.83), and severe nicotine dependence (FTND ≥ 7 points, OR: 2.09, 95% CI: 1.67–2.61). In contrast, these factors showed no significant association with cigar use among women.

**Figure 2:**
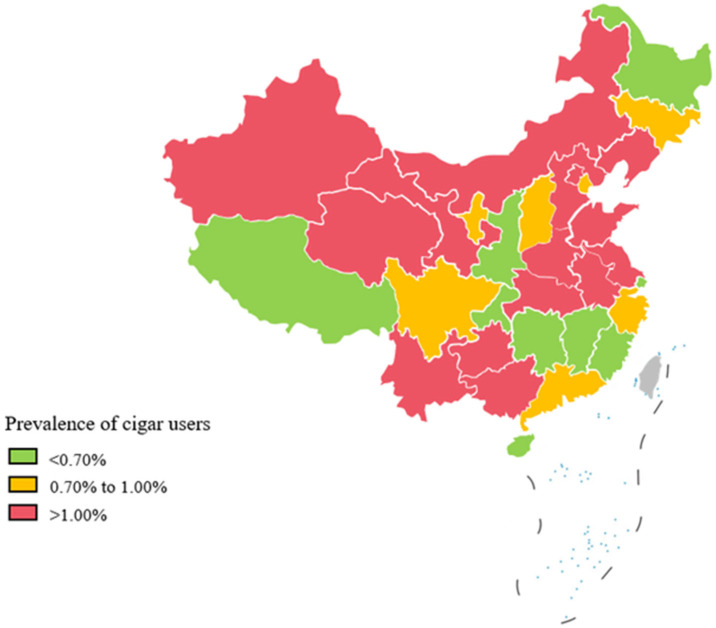
Prevalence of cigar users among the Chinese general population.

The estimated usage rate of cigars among Chinese adults is 1% (95% CI: 1.00–1.01%), with a marked gender disparity: 1.93% (95% CI: 1.92–1.94%) in men and 0.05% (95% CI: 0.04–0.06%) in women. Significant geographic differences were observed, with prevalence rates divided into three categories. The first group, with a prevalence below 0.70%, included provinces like Shanghai (0.14%), Chongqing (0.26%), and Jiangxi (0.34%). The second group, with moderate prevalence between 0.70% and 1.00%, included Shanxi (0.84%), Tianjin (0.95%), and Ningxia (0.99%). The third group, with rates exceeding 1.00%, included regions like Shandong (2.06%) and Inner Mongolia (2.47%) Group differences are depicted in Figure 2.

Overall, we estimated that 10.46 million Chinese adults currently use cigars (95% CI: 10.40–10.53 million), with men accounting for the majority (10.22 million; 95% CI: 10.17–10.26 million). A notable urban–rural disparity was observed, with 3.75 million cigar users in urban areas (95% CI: 3.71–3.78 million) and 6.72 million in rural areas (95% CI: 6.67–6.77 million). The highest number of cigar users was concentrated in the 40–49 age group, totaling 2.39 million (95% CI: 2.36–2.42 million). The results are depicted in Appendix A Table A3.

## 4. Discussion

To the best of our knowledge, this study provides the first comprehensive examination of cigar use in China, revealing a unique demographic profile characterized by individuals who are “knowledgeable but economically marginalized” (KEM). This term captures cigar users with higher educational attainment but lower economic status, a group that deviates from conventional tobacco user profiles in the existing literature.

### 4.1. Key Findings and Comparisons

Our analysis found that cigar prevalence among Chinese adults is relatively low at 1.00%. However, the estimated number of cigar users exceeds 10 million, highlighting a significant public health concern. The findings align with international studies indicating that cigars, although less prevalent than cigarettes, still represent a growing tobacco consumption trend in certain populations.

Compared with the United States, where 6.8% of adults have used cigars [22], China’s cigar users are predominantly men with higher education but lower incomes. This contrasts with the stereotype of cigars being luxury items consumed by affluent individuals. In Finland, cigar use reaches 20.51%, one of the highest rates in Europe, while in Ireland, it is only 1.15% [6], highlighting stark regional disparities influenced by cultural norms, economic conditions, and tobacco control policies. Similarly, in Argentina, 7.8% of youth report using cigars [23], driven by cultural and social factors such as indigenous heritage, socio-economic constraints, and peer influence. In Qatar, only 0.7% of the population uses cigars [24], likely reflecting stringent tobacco control measures and socio-economic factors limiting use. These international comparisons reveal that cigar consumption is shaped by a complex interplay of marketing strategies, cultural acceptance, economic status, and regulatory frameworks, suggesting that the aspirational image of cigars often misaligns with the reality of users’ socio-economic profiles worldwide.

### 4.2. Socioeconomic Paradox: Knowledge vs. Economic Marginalization

In this study, we found that individuals with an annual household income below 20,000 yuan and an education level of junior high school or above constitute a high-risk group for cigar use. We define this group with specific characteristics as the “knowledgeable yet economically marginalized group”. The formation of this phenomenon may stem from targeted cigar marketing strategies that portray cigars as symbols of success, sophistication, and rebellion [25,26]. Such marketing tactics often appeal to individuals with higher education levels, who are more likely to encounter and internalize these aspirational narratives, despite their limited economic means [27]. This phenomenon highlights the complex and often contradictory role of education in tobacco use behaviors.

Education is conventionally viewed as a protective factor against harmful behaviors, including smoking, due to its association with increased health literacy and awareness of the risks associated with tobacco use. However, in the case of cigar smoking, higher education may paradoxically increase susceptibility to marketing strategies that associate cigar use with social prestige, sophistication, and individuality [28]. Individuals in the KEM group might view cigars as attainable symbols of upward mobility or rebellion against economic constraints, leading them to prioritize perceived social benefits over financial and health considerations.

Furthermore, the interplay between marketing and social pressures cannot be overlooked [29]. In professional or social environments where cigar use is glamorized, individuals with higher education but limited economic resources may feel compelled to adopt this behavior to project an image of success or conformity to elite standards. This dynamic is particularly concerning as it not only undermines the health advantages typically associated with education but also perpetuates inequities in tobacco-related harm within this vulnerable demographic.

### 4.3. Regional Variations in Cigar Use

Our study revealed significant geographic variations in cigar use across China, driven by distinct regional factors specific to cigar consumption patterns. Regions with lower cigar prevalence, typically urbanized and economically developed areas, likely benefit from stricter tobacco control policies, higher health literacy, and greater access to smoking cessation resources [30]. These factors may reduce the appeal of cigars in such areas despite the availability of other tobacco products.

Moderate-prevalence regions reflect a more complex interplay of socioeconomic and cultural dynamics. Economic development may increase disposable income, making cigars more accessible, while traditional social norms around tobacco use may persist [31]. The presence of regional tobacco industry operations and marketing efforts could further reinforce cigar consumption.

In higher-prevalence regions, specific factors linked directly to cigar use become evident. These include culturally ingrained smoking practices, the symbolic association of cigars with masculinity or social status, and aggressive local marketing by tobacco companies [32]. Limited public health infrastructure and weaker enforcement of tobacco control policies in such regions may exacerbate the problem.

### 4.4. Policy and Public Health Implications

Given the distinct profile of cigar users in China, current tobacco control policies may need to be adapted. Public health campaigns should address the misconceptions fueled by cigar marketing and target educated but economically constrained individuals, emphasizing the health risks of cigar use regardless of perceived social status.

Moreover, tighter regulations on cigar advertising, particularly in digital and print media, could help reduce the appeal of cigars as symbols of success [29]. Additionally, integrating tobacco cessation services into existing healthcare infrastructure could mitigate the health risks among this vulnerable group [33].

### 4.5. Study Strengths and Limitations

This study benefits from a nationally representative sample and robust data collection methods. However, its cross-sectional design limits causal inferences, and reliance on self-reported data may introduce recall bias. Future longitudinal studies could explore causal pathways linking socioeconomic status, educational attainment, and cigar use. In addition, although our study employed a multistage stratified sampling design to ensure national representation, rural areas were slightly oversampled (54% in our sample compared to approximately 40% in the 2020 Chinese census). Future studies should consider alternative sampling strategies to further optimize representativeness. Additionally, the relatively small number of female cigar users in our sample limited our ability to conduct gender-specific analyses. Future research should focus on increasing female representation to better understand gender-related differences in cigar use patterns.

## 5. Conclusions

This study introduces the concept of the “knowledgeable but economically marginalized” (KEM) group, challenging traditional assumptions about cigar users. The KEM group highlights the complex relationship between socioeconomic inequality and health behaviors, revealing that identifying high-risk populations requires more than just education level or income. The findings suggest that KEM individuals are driven to cigar use by economic pressure, social status aspirations, and insufficient health risk awareness.

To address these issues, public health policies should focus on targeted interventions that combine education and economic context, providing tailored smoking cessation advice. Policymakers should also offer economic support and social welfare to alleviate financial stress and reduce health inequalities. Additionally, regulating tobacco advertising and providing culturally relevant cessation support can help mitigate market misinformation and psychological barriers.

In summary, this study offers new insights into KEM characteristics and provides a direction for public health policy. By integrating targeted identification, socioeconomic support, cultural interventions, and cross-sector collaboration, we can effectively reduce the public health impact of cigar use and promote health equity.

## Figures and Tables

**Table 1:** Baseline characteristics of study participants.

Variables	Used Cigar (n = 1025)			Used Tobacco Product Other than Cigar (n = 21,903)	Never Used Any Tobacco Product (n = 63,773)	Overall (n = 86,701)
	Cigar Only (n = 248)	Cigar and Other Tobacco Product (n = 777)	Overall (n = 1025)			
Gender						
Men	233 (94.0)	750 (96.5)	983 (95.9)	20,695 (94.5)	19,696 (30.9)	41,374 (47.7)
Women	15 (6.0)	27 (3.5)	42 (4.1)	1208 (5.5)	44,077 (69.1)	45,327 (52.3)
Age (%)						
<29	31 (12.5)	64 (8.2)	95 (9.3)	1565 (7.2)	7962 (12.5)	9622 (11.1)
30–39	26 (10.5)	111 (14.3)	137 (13.4)	3019 (13.8)	10,919 (17.1)	14,075 (16.2)
40–49	46 (18.5)	197 (25.4)	243 (23.6)	5239 (23.9)	15,175 (23.8)	20,657 (23.8)
50–59	66 (26.6)	221 (28.4)	287 (28.0)	6295 (28.7)	15,841 (24.8)	22,423 (25.9)
60–69	79 (31.9)	184 (23.7)	263 (25.7)	5785 (26.4)	13,876 (21.8)	19,924 (23.0)
Mean age (SD)	48.9 (13.8)	49.4 (12.3)	49.3 (12.5)	49.9 (12.0)	47.0 (13.4)	47.7 (13.1)
Urbanization, %						
Urban	102 (41.1)	382 (49.2)	484 (47.2)	9431 (43.1)	29,568 (46.4)	39,483 (45.5)
Rural	146 (58.9)	395 (50.8)	541 (52.8)	12,472 (56.9)	34,205 (53.6)	47,218 (54.5)
Ethnicity, %						
Han	215 (86.7)	711 (91.5)	926 (90.3)	19,708 (90.0)	56,016 (87.8)	76,650 (88.4)
Others	33 (13.3)	65 (8.5)	98 (9.7)	2172 (10.0)	7672 (12.2)	9942 (11.6)
Marriage status, %						
Single	29 (11.7)	66 (8.5)	77 (7.7)	1595 (7.3)	5205 (8.2)	6895 (8.0)
Married	201 (81.0)	646 (83.1)	841 (83.7)	18,611 (85.0)	53,956 (84.6)	73,414 (84.6)
Separated/divorced/widowed	17 (6.9)	65 (8.4)	87 (8.6)	1683 (7.7)	4563 (7.2)	6327 (7.4)
Education level, %						
Primary school or less	105 (42.3)	198 (25.5)	95 (9.3)	7369 (33.6)	23,031 (36.1)	30,703 (35.4)
Middle and high school	129 (52.0)	436 (56.1)	847 (82.6)	12,087 (55.2)	30,756 (48.2)	43,408 (50.1)
College and higher	14 (5.6)	143 (18.4)	81 (8.1)	2410 (11.2)	9827 (15.7)	12,392 (14.5)
Annual family income (RMB/year), %						
<20,000	114 (46.0)	231 (29.7)	345 (33.7)	6681 (30.5)	18,183 (28.5)	25,209 (29.1)
20,000–49,999	70 (28.2)	242 (31.3)	312 (30.4)	7389 (33.7)	21,705 (34.0)	29,406 (33.9)
≥50,000	63 (25.4)	303 (39.0)	366 (35.9)	7782 (35.5)	23,674 (37.5)	31,822 (37.0)
Self-reported health status, %						
Good	135 (54.4)	416 (53.5)	551 (53.8)	12,473 (56.9)	37,156 (58.3)	50,180 (57.9)
Average	91 (36.7)	284 (36.6)	375 (36.6)	7778 (35.6)	21,727 (34.1)	29,880 (34.5)
Poor	22 (8.9)	77 (9.9)	96 (9.6)	1586 (7.5)	4635 (7.6)	6317 (7.6)
FTND						
0–3	126 (50.8)	328 (42.2)	454 (44.3)	11,663 (53.2)	-	11,663 (53.2)
4–6	101 (40.7)	360 (46.3)	461 (45.0)	8712 (39.8)	-	8712 (39.8)
≥7	21 (8.5)	89 (11.5)	110 (10.7)	1469 (7.0)	-	1469 (7.0)
Mean score (SD)	3.4 (2.0)	3.9 (2.1)	3.8 (2.1)	3.4 (2.0)	-	-

Note: SD: Standard deviation; FTND: Fagerstrom test for nicotine data is shown as number (%) or mean (SD). The differing denominators used in the calculation of percentages are because of missing data.

**Table 2:** Adjusted ORs for cigar use associated with baseline information among cigar users and users of tobacco products other than cigars.

Variable	Men		Women	
	OR (95% CI)	*p*	OR (95% CI)	*p*
Age				
<29	Ref		Ref	
30–39	0.75 (0.56, 1.10)	0.52	2.06 (0.46, 9.17)	0.34
40–49	0.79 (0.69, 1.05)	0.83	2.02 (0.44, 9.21)	0.36
50–59	0.78 (0.59, 1.04)	0.57	1.14 (0.23, 5.62)	0.87
60–69	0.80 (0.60, 1.07)	0.77	1.10 (0.22, 5.6)	0.91
Urbanization				
Urban	Ref		Ref	
Rural	0.89 (0.78, 1.02)	0.15	0.52 (0.26, 1.05)	0.07
Ethnicity				
Other	Ref		Ref	
Han	1.101 (0.88, 1.38)	0.09	2.13 (0.72, 6.29)	0.17
Marriage status				
Separated/divorced/widowed	Ref		Ref	
Married	1.06 (0.76, 1.49)	0.23	0.92 (0.35, 2.44)	0.86
Single	0.98 (0.77, 1.25)	0.18	1.79 (0.33, 9.76)	0.50
Education level				
Primary school or less	Ref		Ref	
Middle and high school	1.23 (1.05, 1.44)	<0.0001 ***	0.75 (0.34, 1.65)	0.47
College and higher	1.81 (1.42, 2.30)	0.03 *	0.49 (0.14, 1.66)	0.25
Annual family income (RMB/year)				
>49,999	Ref		Ref	
20,000–49,999	1.28 (0.87, 1.21)	0.99	0.60 (0.26, 1.40)	0.24
<20,000	1.02 (1.08, 1.53)	0.03 *	0.92 (0.40, 2.12)	0.84
Self-reported health status				
Good	Ref		Ref	
Average	1.12 (0.98, 1.29)	0.32	0.77 (0.39, 1.51)	0.44
Poor	1.45 (1.15, 1.83)	<0.0001 ***	0.51 (0.15, 1.79)	0.30
FTND				
0–3	Ref		Ref	
4–6	1.43 (1.25, 1.64)	<0.0001 ***	2.74 (1.39, 5.40)	0.003 **
≥7	2.09 (1.67, 2.61)	<0.0001 ***	3.34 (0.91, 12.21)	0.07

Note: Model was adjusted for gender, age, urbanization, ethnicity, marriage status, education level, annual family income, self-reported health status, and FTND score. OR: Odd ratio; CI: Confidence interval; Ref: Reference. *p*-values less than 0.0001 are presented as “*p* < 0.0001”, while those greater than 0.0001 but less than 0.05 are shown with their exact values. Asterisks indicate levels of statistical significance: * *p* < 0.05, ** *p* < 0.01, *** *p* < 0.001.

## Data Availability

Data will be available upon reasonable request to the corresponding author immediately following publication to anyone wishing to access the data.

## Appendix A

**Table A1:** Comparison of the demographic characteristics and health behaviors between cigar users and non-tobacco product users.

Variables	Used Cigars (n = 1025)	Never Used Any Tobacco Products (n = 63,773)	*p* ^#^
Gender			<0.0001 ***
Men	983 (95.9)	19,696 (30.9)	
Women	42 (4.1)	44,077 (69.1)	
Age (%)			<0.0001 ***
<29	95 (9.3)	7962 (12.5)	
30–39	137 (13.4)	10,919 (17.1)	
40–49	243 (23.6)	15,175 (23.8)	
50–59	287 (28.0)	15,841 (24.8)	
60–69	263 (25.7)	13,876 (21.8)	
Mean age (SD)	49.3 (12.5)	47.0 (13.4)	
Urbanization, %			<0.0001 ***
Urban	484 (47.2)	29,568 (46.4)	
Rural	541 (52.8)	34,205 (53.6)	
Ethnicity, %			0.018
Han	926 (90.3)	56,016 (87.8)	
Others	98 (9.7)	7672 (12.2)	
Marriage status, %			0.764
Single	77 (7.7)	5205 (8.2)	
Married	841 (83.7)	53,956 (84.6)	
Separated/divorced/widowed	87 (8.6)	4563 (7.2)	
Education level, %			0.001 **
Primary school or less	95 (9.3)	23,031 (36.1)	
Middle and high school	847 (82.6)	30,756 (48.2)	
College and higher	81 (8.1)	9827 (15.7)	
Annual family income (RMB/year), %			0.013 *
<20,000	345 (33.7)	18,183 (28.5)	
20,000–49,999	312 (30.4)	21,705 (34.0)	
≥50,000	366 (35.9)	23,674 (37.5)	
Self-reported health status, %			0.001 **
Good	551 (53.8)	37,156 (58.3)	
Average	375 (36.6)	21,727 (34.1)	
Poor	96 (9.6)	4635 (7.6)	
FTND		-	-
0–3	454 (44.3)	-	
4–6	461 (45.0)	-	
≥7	110 (10.7)	-	
Mean score (SD)	3.8 (2.1)		

Note: SD: Standard deviation; FTND: Fagerstrom test for nicotine data is shown as number (%) or mean (SD). The differing denominators used in the calculation of percentages are because of missing data. # *p* value for difference between group who have used cigars and group who have never used any tobacco product. *p*-values less than 0.0001 are presented as “*p* < 0.0001”, while those greater than 0.0001 but less than 0.05 are shown with their exact values. Asterisks indicate levels of statistical significance: * *p* < 0.05, ** *p* < 0.01, *** *p* < 0.001.

**Table A2:** Prevalence of cigar use in different annual household income and education levels among Chinese adults.

Age (Year)		<29	30–39	40–49	50–59	60–69	Total
Men	Annual family income (RMB/year)						
	<20,000	1.76%	2.39%	2.66%	2.93%	2.77%	2.68%
	20,000–49,999	1.68%	1.83%	2.09%	2.42%	2.43%	2.19%
	>49,999	2.63%	2.10%	2.43%	2.30%	2.13%	2.30%
	Education level						
	Primary school or less	0.67%	1.14%	2.39%	2.37%	2.38%	2.25%
	Middle and high school	2.13%	2.26%	2.28%	2.54%	2.66%	2.41%
	College and higher	2.55%	2.19%	2.62%	3.25%	1.84%	2.49%
Women	Annual family income (RMB/year)						
	<20,000	0.27%	0.07%	0.14%	0.11%	0.10%	0.12%
	20,000–49,999	0.00%	0.04%	0.10%	0.09%	0.03%	0.06%
	>49,999	0.00%	0.15%	0.10%	0.03%	0.18%	0.10%
	Education level						
	Primary school or less	0.25%	0.00%	0.17%	0.09%	0.11%	0.11%
	Middle and high school	0.03%	0.14%	0.07%	0.08%	0.07%	0.08%
	College and higher	0.05%	0.09%	0.08%	0.00%	0.36%	0.08%
Total	Annual family income (RMB/year)						
	<20,000	0.94%	1.12%	1.36%	1.48%	1.47%	1.37%
	20,000–49,999	0.73%	0.79%	1.01%	1.22%	1.26%	1.06%
	>49,999	1.18%	1.03%	1.21%	1.16%	1.19%	1.15%
	Education level						
	Primary school or less	0.43%	0.45%	1.03%	0.96%	1.11%	0.99%
	Middle and high school	0.98%	1.10%	1.20%	1.45%	1.66%	1.30%
	College and higher	1.12%	1.04%	1.38%	1.95%	1.31%	1.25%

**Table A3:** Estimated prevalence and total number of cigar users in China in 2018.

Variable	Prevalence (95% CI)	Total Number (Million, 95% CI)
Age		
<29	1.14% (1.04%, 1.17%)	2.47 (2.42, 2.52)
30–39	1.06% (1.05%, 1.07%)	2.27 (2.24, 2.30)
40–49	1.04% (1.03%, 1.06%)	2.39 (2.36, 2.42)
50–59	1.34% (1.32%, 1.35%)	2.13 (2.11, 2.16)
60–69	1.21% (1.19%, 1.23%)	1.21 (1.19, 1.23)
Gender		
Men	1.93% (1.92%, 1.94%)	10.22 (10.17, 10.26)
Women	0.05% (0.04%, 0.06%)	0.25 (0.20, 0.29)
Locality		
Urban	1.11% (1.10%, 1.12%)	3.75 (3.71, 3.78)
Rural	0.95% (0.94%, 0.96%)	6.72 (6.67, 6.77)
Overall	1.00% (1.00%, 1.01%)	10.46 (10.40, 10.53)

Note: CI: Confidence interval.
